# Dietary Fiber Intake and Phenotypic Age Acceleration in United States Adults: A Nationally Representative Cross-Sectional Study

**DOI:** 10.7759/cureus.117915

**Published:** 2026-10-14

**Authors:** Keemaya G Joglekar

**Affiliations:** 1 Research, Independent Researcher, Mumbai, IND

**Keywords:** biological aging, dietary fiber, nhanes, phenotypic age, systemic immune-inflammation index, systemic inflammation

## Abstract

Background: Biological age reflects physiological aging beyond chronological age and predicts morbidity and mortality. Higher dietary fiber intake has been linked to slower biological aging, but whether this reflects lower systemic inflammation or improved insulin resistance is unclear. The primary aim was to estimate the association between dietary fiber intake and phenotypic age acceleration among United States adults; a secondary, exploratory aim was to examine whether systemic inflammation or insulin resistance statistically accounts for this association.

Methods: This cross-sectional study analyzed 3,896 adults aged 20 years and older from the National Health and Nutrition Examination Survey (NHANES) 2017-2018 cycle. Phenotypic age was computed from nine clinical chemistry biomarkers plus chronological age; phenotypic age acceleration (PhenoAgeAccel) was defined as the residual from regressing phenotypic age on chronological age. Dietary fiber was assessed by a 24-hour recall. Survey-weighted linear regression (Day 1 dietary weights, strata, and primary sampling units) was adjusted for age, sex, race/ethnicity, body mass index, income, energy intake, smoking, and physical activity. As an exploratory secondary analysis, the association was decomposed using the product-of-coefficients method with the systemic immune-inflammation index (SII) and, in the fasting subsample, HOMA-IR (Homeostatic Model Assessment of Insulin Resistance) as candidate mediators.

Results: Each additional 10 g/day of dietary fiber was associated with a 0.66-year lower PhenoAgeAccel (95% CI -0.92 to -0.40; p<0.001) in the fully adjusted model, with a graded dose-response; the association was unchanged after excluding extreme values and did not differ by sex or race/ethnicity. In the exploratory analysis, systemic inflammation statistically accounted for 17.5% of the association (indirect effect -0.115; 95% CI -0.203 to -0.017); however, because the inflammation index shares leukocyte-derived components with the phenotypic age algorithm, a sensitivity analysis removing these shared components attenuated the indirect effect to -0.045, which accounted for 8.7% of the total effect estimated in that sensitivity model. Insulin resistance showed no statistically detectable indirect association in the fasting subsample.

Conclusions: Higher dietary fiber intake was associated with lower phenotypic age acceleration in a nationally representative sample of United States adults. Systemic inflammation statistically accounted for a modest share, but the exploratory estimate is roughly halved once shared measurement is accounted for and cannot be interpreted causally given the cross-sectional design. These findings identify dietary fiber as a low-cost, broadly accessible target for prospective and interventional studies of healthy aging.

## Introduction

Chronological age is the strongest risk factor for most noncommunicable diseases, yet individuals of the same chronological age vary widely in physiological condition [1]. Composite “biological age” measures capture this heterogeneity. Phenotypic Age, derived from nine routine clinical chemistry biomarkers together with chronological age, is among the most extensively validated; it predicts all-cause and cause-specific mortality across diverse subpopulations and is computable from standard blood panels [2]. The residual between Phenotypic Age and chronological age, termed phenotypic age acceleration (PhenoAgeAccel), summarizes whether a person is aging faster or slower than expected [2].

Chronic low-grade inflammation (“inflammaging”) accompanies advancing age and contributes to age-related disease and frailty [3]. Diet is among the most modifiable determinants of inflammation and health [4]. Dietary fiber has well-established metabolic benefits, including improved glycemic control, favorable lipid profiles, lower body weight, reduced inflammatory markers, and lower premature mortality [5]. Fermentable fiber is metabolized by the gut microbiota into short-chain fatty acids with systemic anti-inflammatory and immunoregulatory effects [6], providing a plausible biological link between fiber and slower aging.

Recent cross-sectional analyses have linked higher intake of several nutrients, including fiber, to lower phenotypic age acceleration [7], but without formally examining the pathways involved. This study therefore had a primary aim to estimate the association between dietary fiber intake and phenotypic age acceleration among United States adults, and to test whether it varies by sex or race/ethnicity, and a secondary, exploratory aim to examine whether systemic inflammation or insulin resistance statistically accounts for the association. It was hypothesized that higher dietary fiber intake would be associated with lower phenotypic age acceleration, and that part of that association would be statistically attributable to lower systemic inflammation. Given the cross-sectional design, the exploratory analysis was interpreted as a decomposition of the observed association rather than as evidence of a proven causal mechanism.

## Materials and methods

Study design and population

This cross-sectional study used data from the National Health and Nutrition Examination Survey (NHANES) 2017-2018 cycle, a nationally representative, complex multistage probability survey of the civilian, noninstitutionalized United States population conducted by the National Center for Health Statistics (NCHS) [8]. Adults aged 20 years and older with a computable Phenotypic Age and valid dietary fiber data were eligible. Of 4,894 eligible adults, 3,896 (80%) had complete data on all covariates and constituted the analytic sample; a complete-case approach was used. Excluded participants were slightly older than those included (mean 53.0 vs. 51.1 years). Insulin resistance analyses were restricted to the morning fasting subsample. The participant selection process is summarized in Figure 1.

**Figure 1:**
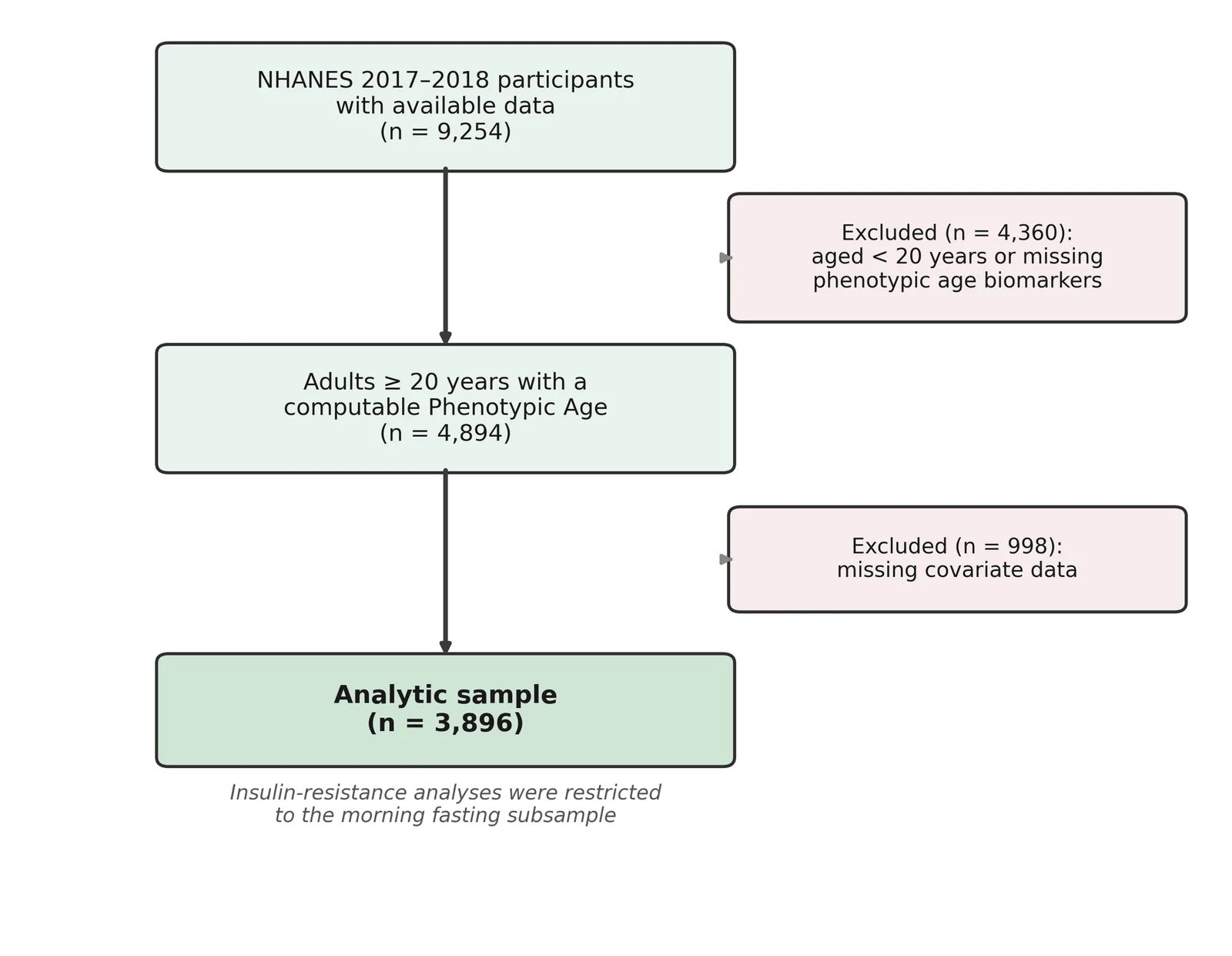
Participant flow diagram, NHANES 2017-2018 The figure was generated using Python 3.11 (Python Software Foundation, Wilmington, DE, USA) with the Matplotlib library. NHANES, National Health and Nutrition Examination Survey

Dietary fiber assessment

Total dietary fiber (grams/day) was obtained from the first 24-hour dietary recall (variable DR1TFIBE), coded using the United States Department of Agriculture Food and Nutrient Database for Dietary Studies. Fiber was modeled continuously and scaled per 10 g/day; quintiles were used to describe the dose-response relationship.

Phenotypic age and phenotypic age acceleration

Phenotypic Age was calculated using the validated algorithm of Liu et al. [2] from chronological age and nine clinical chemistry biomarkers: albumin, creatinine, serum glucose, C-reactive protein, lymphocyte percentage, mean corpuscular volume, red-cell distribution width, alkaline phosphatase, and white-blood-cell count. Following standard practice, phenotypic age acceleration (PhenoAgeAccel) was defined as the residual from a linear regression of Phenotypic Age on chronological age, so that positive values indicate faster-than-expected aging that is statistically independent of chronological age.

Candidate mediators

Systemic inflammation was operationalized as the systemic immune-inflammation index (SII=platelet count×neutrophil count/lymphocyte count), first proposed by Hu et al. [9] and subsequently validated as a prognostic inflammatory index [10]; it was natural-log transformed. The SII shares leukocyte-derived components (lymphocytes and neutrophils) with the Phenotypic Age algorithm, which includes lymphocyte percentage and white-blood-cell count; this overlap is examined in a sensitivity analysis (below). C-reactive protein was not used as a mediator because it is itself a component of Phenotypic Age; using it would introduce direct mathematical overlap with the outcome and could artificially inflate the estimated mediation. Insulin resistance was quantified in the fasting subsample using Homeostatic Model Assessment of Insulin Resistance (HOMA-IR) (fasting glucose (mg/dL)×fasting insulin (µU/mL)/405), as described by Matthews et al. [11], and natural-log transformed.

Covariates

Models were adjusted for chronological age (years), sex, race/ethnicity, body mass index (kg/m²), family income-to-poverty ratio, total energy intake (kcal/day), smoking status (never, former, or current), and any recreational physical activity (yes/no). Race/ethnicity was modeled using the six categories (non-Hispanic White, non-Hispanic Black, non-Hispanic Asian, Mexican American, other Hispanic, and other/multiracial). Smoking status was modeled as a three-level categorical variable (never, former, current), and recreational physical activity as a binary indicator.

Statistical analysis

All analyses incorporated the NHANES complex survey design following NCHS analytic guidelines [8], using the Day 1 dietary two-year sample weight (WTDRD1), appropriate because dietary fiber was derived from the Day 1 24-hour recall, together with the design strata (SDMVSTRA) and primary sampling units (SDMVPSU); variances were estimated using design-based cluster-robust (sandwich) standard errors with primary sampling units as the clustering unit. The association between fiber and PhenoAgeAccel was estimated using survey-weighted linear regression across a hierarchy of progressively adjusted models, and its robustness was assessed by excluding participants with extreme acceleration (|PhenoAgeAccel|>3 standard deviations). As an exploratory secondary analysis, the total association was decomposed by the product-of-coefficients method into direct and indirect (mediator-associated) components; 95% confidence intervals for indirect effects were obtained from a cluster bootstrap resampling of primary sampling units (1,000 iterations). Because mediation from cross-sectional data cannot establish temporal ordering, indirect effects are interpreted as statistical decomposition rather than as causal pathways. To assess whether the inflammation-associated component reflected shared measurement, a prespecified sensitivity analysis recomputed Phenotypic Age with the shared leukocyte components (white-blood-cell count and lymphocyte percentage) held at their sample means. Effect modification by sex and race/ethnicity was tested using multiplicative interaction terms with a joint Wald test. A two-sided p<0.05 was defined as significance. All analyses were performed in 2026 using Python 3.11.15 (Python Software Foundation, Wilmington, DE, USA) with the pandas (v3.0.2; The pandas development team, Worldwide), statsmodels (v0.14.6; statsmodels development team, Worldwide), NumPy (v2.4.4; NumPy Developers, Worldwide), and SciPy (v1.17.1; SciPy Developers, Worldwide) libraries. No formal correction for multiplicity was applied; the progressively adjusted models, the sex and race/ethnicity interaction tests, and the sensitivity analyses were specified in advance and are reported in full, and the secondary and exploratory results should therefore be read as hypothesis-generating rather than confirmatory.

## Results

Participant characteristics

The analytic sample comprised 3,896 adults aged 20 years and older (Table 1). The mean age was 51.1 years, and 1,996 participants (51.2%) were women. Mean dietary fiber intake was 16.8 g/day, below recommended levels, and the mean body mass index was 29.9 kg/m².

**Table 1:** Characteristics of the analytic sample, NHANES 2017-2018 (n=3,896) Continuous variables are presented as mean (SD); categorical variables are presented as unweighted count (unweighted percentage). This descriptive table is unweighted and therefore characterizes the analytic sample rather than the United States population. No hypothesis tests were performed for this table, and no test statistics are reported. SD, standard deviation; n, number of participants; NHANES, National Health and Nutrition Examination Survey

Characteristic	Value
Participants, n	3,896
Age, years, mean (SD)	51.1 (17.4)
Women, n (%)	1,996 (51.2)
Men, n (%)	1,900 (48.8)
Dietary fiber, g/day, mean (SD)	16.8 (10.9)
Energy intake, kcal/day, mean (SD)	2,125 (1,043)
Body mass index, kg/m^2^, mean (SD)	29.9 (7.4)
Non-Hispanic White, n (%)	1,489 (38.2)
Non-Hispanic Black, n (%)	852 (21.9)
Mexican American, n (%)	503 (12.9)
Non-Hispanic Asian, n (%)	500 (12.8)
Other Hispanic, n (%)	346 (8.9)
Other/multiracial, n (%)	206 (5.3)
Never smoker, n (%)	2,199 (56.4)
Former smoker, n (%)	991 (25.4)
Current smoker, n (%)	706 (18.1)
Physically active, n (%)	1,852 (47.5)
Phenotypic Age, years, mean (SD)	50.4 (20.5)

Dietary fiber and biological age acceleration (primary analysis)

Higher fiber intake was associated with lower PhenoAgeAccel in a graded, dose-response fashion (Figure 2).

**Figure 2:**
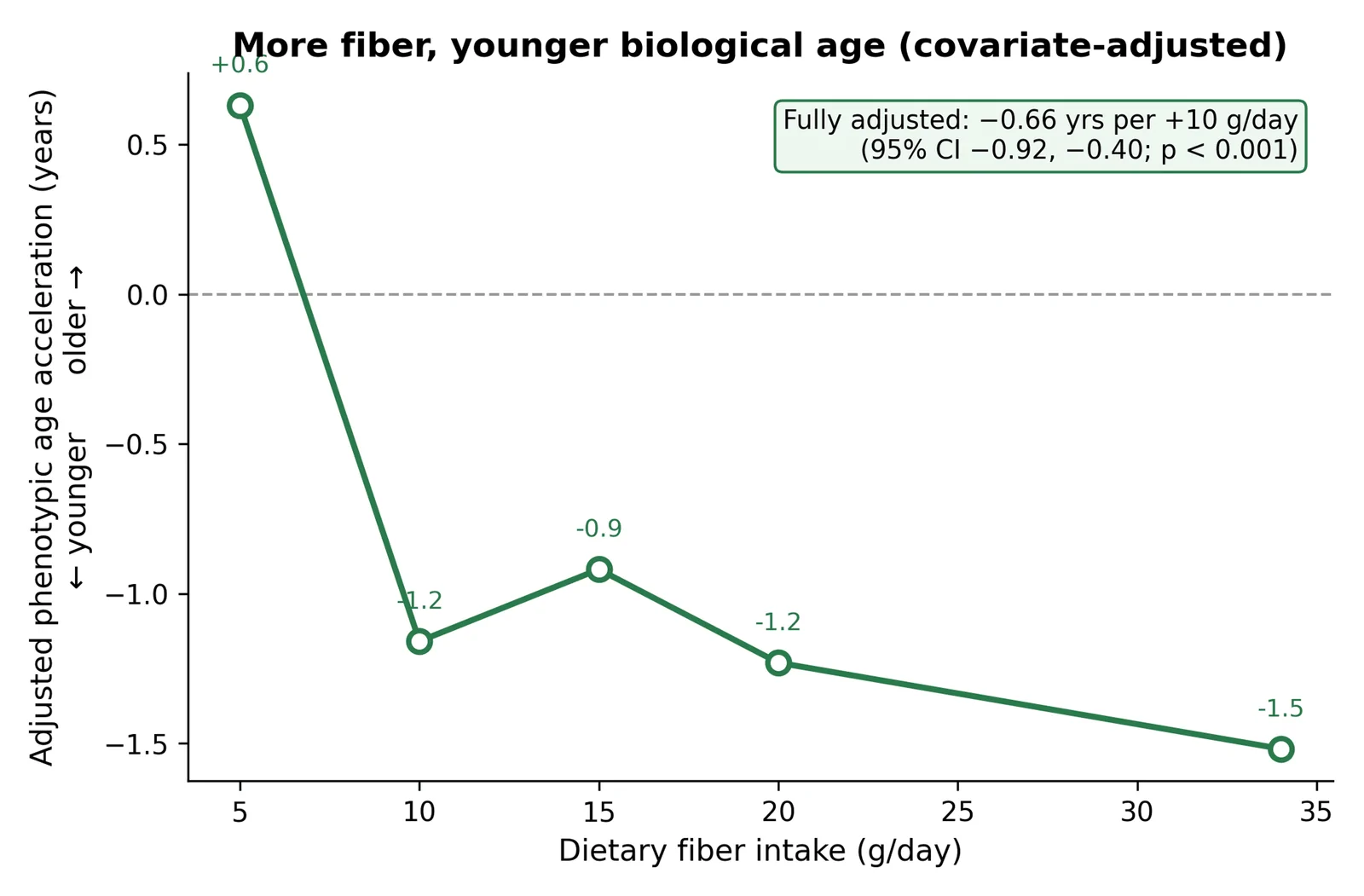
Dietary fiber intake and phenotypic age acceleration Covariate-adjusted marginal mean phenotypic age acceleration across quintiles of dietary fiber intake, NHANES 2017-2018. Points are model-based adjusted means from survey-weighted regression; the annotated estimate is the fully adjusted association per 10 g/day of fiber. The figure was generated using Python 3.11 (Python Software Foundation, Wilmington, DE, USA) with the Matplotlib library. NHANES, National Health and Nutrition Examination Survey

In the fully adjusted, survey-weighted model, each additional 10 g/day of fiber was associated with a 0.66-year lower phenotypic age (95% CI -0.92 to -0.40; p=5.5×10⁻⁷). The association was consistent across a hierarchy of progressively adjusted models (Table 2) and was materially unchanged after excluding participants with extreme acceleration values (|PhenoAgeAccel|>3 SD; β=-0.70 per 10 g/day; p<0.001). It did not differ significantly by sex (interaction p=0.68) or race/ethnicity (joint interaction p=0.83), indicating a broadly consistent relationship.

**Table 2:** Association of dietary fiber intake with phenotypic age acceleration across progressively adjusted survey-weighted linear regression models Estimates are regression coefficients (β), expressed as years of phenotypic age acceleration per additional 10 g/day of dietary fiber, with 95% CI. Test statistic: survey-weighted linear regression with design-based cluster-robust variance estimation, using the Day 1 dietary sample weight (WTDRD1), strata, and primary sampling units. Each model adds the listed covariates to those of the preceding model.

Model	Covariates added	Beta (years per 10 g/day)	95% CI	p-value
Model 1	Unadjusted	-0.85	-1.14 to -0.56	<0.001
Model 2	Age, sex, race/ethnicity	-0.93	-1.25 to -0.61	<0.001
Model 3	Model 2 plus body mass index, income-to-poverty ratio, total energy intake	-0.83	-1.09 to -0.58	<0.001
Model 4	Model 3 plus smoking status, physical activity	-0.66	-0.92 to -0.40	<0.001

Exploratory decomposition by inflammation and insulin resistance

In an exploratory analysis, systemic inflammation statistically accounted for a modest share of the association (Table 3): the indirect (inflammation-associated) effect was -0.115 years per 10 g/day (95% CI -0.203 to -0.017), or 17.5% of the total effect. However, because the inflammation index shares leukocyte-derived components with the phenotypic age algorithm, a prespecified sensitivity analysis that removed these shared components attenuated the indirect effect to -0.045 years (95% CI -0.079 to -0.011; 8.7% of the total), roughly half of the primary estimate, although it remained statistically significant. This indicates that a substantial portion of the apparent inflammatory contribution reflects shared measurement rather than an independent pathway; the residual inflammatory association is small, and the finding should be interpreted cautiously. Because exposure, mediator, and outcome were measured concurrently, these estimates should be read as statistical mediation consistent with, but not establishing, a mediating pathway. Insulin resistance did not contribute to the association in the fasting subsample (indirect effect not distinguishable from zero).

**Table 3:** Exploratory decomposition of the association between dietary fiber intake and phenotypic age acceleration by systemic inflammation Effects are expressed in years of phenotypic age acceleration per additional 10 g/day of dietary fiber. Systemic inflammation was operationalized as the log-transformed SII. Test statistic: indirect effects were estimated by the product-of-coefficients method, with 95% CI derived from cluster bootstrap resampling of primary sampling units (1,000 resamples for the primary estimate; 500 for the sensitivity estimate). The sensitivity analysis recomputes Phenotypic Age with the leukocyte-derived components shared with the SII (white blood cell count and lymphocyte percentage) held constant. c, total effect; c', direct effect; a×b, indirect effect; II, systemic immune-inflammation index.

Effect	Notation	Estimate (years per 10 g/day)	95% CI	Percentage of total effect
Total effect	c	-0.659	-0.916 to -0.401	100.0
Direct effect	c'	-0.543	-0.789 to -0.298	82.5
Indirect effect via systemic inflammation, primary analysis	axb	-0.115	-0.203 to -0.017	17.5
Indirect effect via systemic inflammation, sensitivity analysis	axb	-0.045	-0.079 to -0.011	8.7

## Discussion

In a nationally representative sample of United States adults, higher dietary fiber intake was associated with a younger phenotypic age. The association followed a clear dose-response pattern, persisted across progressively adjusted models, was unchanged after excluding extreme values, and was consistent across sex and racial/ethnic groups. This primary finding is the central contribution of the study and extends prior cross-sectional evidence linking fiber to phenotypic age [7].

The exploratory decomposition provides only tentative support for systemic inflammation as an explanatory pathway, and two considerations temper its interpretation. First, the cross-sectional design cannot establish that fiber intake preceded differences in inflammation or aging; reverse causation is possible, and the decomposition is best regarded as descriptive rather than causal. Second, the inflammation index shares leukocyte-derived components with the phenotypic age measure, and accounting for this overlap roughly halved the inflammation-associated proportion (from 17.5% of the primary total effect to 8.7% of the total effect estimated in the sensitivity model). Inflammation may therefore account statistically for a modest share, consistent with the anti-inflammatory mechanistic plausibility of dietary fiber [2,4] and with prior work in which inflammatory markers explained only a limited share of diet-aging relationships [12], but the present data cannot isolate it as the mechanism. These findings align with mediation analyses of other aging exposures, in which diet quality partially accounted for the tooth-loss-aging association [13], and inflammatory indices partially accounted for the body-fat-aging association [14], while underscoring the need for cautious interpretation when mediators and outcomes share measurement.

The absence of an insulin-resistance contribution was unexpected given fiber's glycemic benefits in trials [5]; the smaller fasting subsample, a single cross-sectional HOMA-IR measurement, and the composition of Phenotypic Age (weighted toward hepatic, renal, and hematologic markers) may all contribute. The absence of a statistically detectable indirect association does not establish that insulin resistance plays no biological role; it indicates only that none was detected in this smaller fasting subsample. Plausible alternative pathways include fiber's fermentation to short-chain fatty acids, which reinforce intestinal barrier integrity and limit translocation of pro-inflammatory microbial products, together with the promotion of greater gut microbial diversity and favorable modulation of bile-acid metabolism. These mechanisms have each been linked to lower systemic inflammation and slower biological aging [6].

Clinical and public health implications

Biological age measures such as Phenotypic Age are increasingly used to stratify risk and evaluate lifestyle interventions [2]. The observed magnitude, roughly two-thirds of a year of phenotypic age per 10 g/day of fiber, is meaningful at the population level, particularly because mean intake in this sample (16.8 g/day) was well below recommended amounts. Dietary fiber is inexpensive, widely accessible, and free of the adverse effects of pharmacologic anti-inflammatory strategies, making it an attractive candidate for prospective evaluation in healthy-aging research. The consistency of the association across sex and racial/ethnic groups suggests that guidance to increase intake of fiber-rich whole grains, legumes, fruits, and vegetables could benefit the population broadly and equitably [5].

Strengths and limitations

Strengths include a large, nationally representative sample with appropriate survey weighting [8], objective biomarker-based outcomes, comprehensive confounder adjustment, and transparent sensitivity analyses. Limitations include the cross-sectional design, which precludes causal inference and permits reverse causation; assessment of fiber from a single 24-hour recall, which introduces measurement error and likely biases associations toward the null; a complete-case analysis that excluded 20% of eligible adults, who were slightly older; the smaller fasting subsample for insulin resistance; the shared leukocyte components between the SII and Phenotypic Age, which limit interpretation of the inflammatory decomposition; residual confounding from factors we could not fully capture, including alcohol intake, educational attainment, medication use, chronic kidney disease, and overall comorbidity burden; the possibility that overall diet quality rather than fiber specifically drives the association, given that fiber-rich diets track with broader healthy-eating patterns such as a higher Healthy Eating Index [15]; restriction to a single NHANES cycle (2017-2018); fiber obtained from supplements, which is not captured by the 24-hour dietary recall and would misclassify total intake among supplement users; and the absence of any formal correction for multiplicity across the model hierarchy, subgroup tests, and exploratory decomposition. Future studies using repeated 24-hour recalls or multi-day dietary records could better capture habitual intake and reduce exposure misclassification.

## Conclusions

In this nationally representative sample of United States adults, higher dietary fiber intake was associated with lower phenotypic age acceleration, that is, a younger phenotypic age relative to chronological age, following a clear dose-response gradient that persisted after full adjustment, outlier exclusion, and across sex and racial/ethnic groups. An exploratory decomposition indicated that systemic inflammation statistically accounted for a modest share of this association, but the estimate was roughly halved once shared measurement between the inflammation index and the aging measure was accounted for and cannot be interpreted causally given the cross-sectional design. Insulin resistance showed no statistically detectable indirect association in the fasting subsample, which does not establish the absence of a biological role. These findings identify dietary fiber intake as a low-cost, widely accessible, and equitable target for prospective and interventional studies designed to determine whether this association is causal.

## References

[1] Niccoli T, Partridge L (2012). Ageing as a risk factor for disease. Curr Biol.

[2] Liu Z, Kuo PL, Horvath S, Crimmins E, Ferrucci L, Levine M (2018). A new aging measure captures morbidity and mortality risk across diverse subpopulations from NHANES IV: a cohort study. PLoS Med.

[3] Batista MA, Calvo-Fortes F, Silveira-Nunes G (2020). Inflammaging in endemic areas for infectious diseases. Front Immunol.

[4] Minihane AM, Vinoy S, Russell WR (2015). Low-grade inflammation, diet composition and health: current research evidence and its translation. Br J Nutr.

[5] Reynolds AN, Akerman AP, Mann J (2020). Dietary fibre and whole grains in diabetes management: systematic review and meta-analyses. PLoS Med.

[6] Silva YP, Bernardi A, Frozza RL (2020). The role of short-chain fatty acids from gut microbiota in gut-brain communication. Front Endocrinol (Lausanne).

[7] Ma J, Li P, Jiang Y (2024). The association between dietary nutrient intake and acceleration of aging: evidence from NHANES. Nutrients.

[8] Johnson CL, Paulose-Ram R, Ogden CL, Carroll MD, Kruszon-Moran D, Dohrmann SM, Curtin LR (2013). National health and nutrition examination survey: analytic guidelines, 1999-2010. Vital Health Stat 2.

[9] Hu B, Yang XR, Xu Y (2014). Systemic immune-inflammation index predicts prognosis of patients after curative resection for hepatocellular carcinoma. Clin Cancer Res.

[10] Li M, Li Z, Wang Z, Yue C, Hu W, Lu H (2022). Prognostic value of systemic immune-inflammation index in patients with pancreatic cancer: a meta-analysis. Clin Exp Med.

[11] Matthews DR, Hosker JP, Rudenski AS, Naylor BA, Treacher DF, Turner RC (1985). Homeostasis model assessment: insulin resistance and beta-cell function from fasting plasma glucose and insulin concentrations in man. Diabetologia.

[12] Millar CL, Dufour AB, Shivappa N (2022). A proinflammatory diet is associated with increased odds of frailty after 12-year follow-up in a cohort of adults. Am J Clin Nutr.

[13] Xu KH, Li L, Jia SL (2023). Association of tooth loss and diet quality with acceleration of aging: evidence from NHANES. Am J Med.

[14] Bai YS, Yu SX, Xia L (2026). Body fat distribution and aging: unveiling association and potential intervention strategies. Nutrition.

[15] Krebs-Smith SM, Pannucci TE, Subar AF (2018). Update of the Healthy Eating Index: HEI-2015. J Acad Nutr Diet.

